# The prevalence of religiosity and association between religiosity and alcohol use, other drug use, and risky sexual behaviours among grade 8-10 learners in Western Cape, South Africa

**DOI:** 10.1371/journal.pone.0211322

**Published:** 2019-02-13

**Authors:** Joel Msafiri Francis, Bronwyn Myers, Sebenzile Nkosi, Petal Petersen Williams, Tara Carney, Carl Lombard, Elmarie Nel, Neo Morojele

**Affiliations:** 1 Visiting scholar, Alcohol, Tobacco & Other Drug Research Unit, South African Medical Research Council, Pretoria, South Africa; 2 Wits Reproductive Health & HIV Institute (WRHI), University of the Witwatersrand, Johannesburg, South Africa; 3 School of Clinical Medicine, Faculty of Health Sciences, University of the Witwatersrand, Johannesburg, South Africa; 4 Department of Epidemiology and Biostatistics, Muhimbili University of Health and Allied Sciences, Dar es Salaam, Tanzania; 5 Alcohol, Tobacco & Other Drug Research Unit, South African Medical Research Council, Tygerberg, Cape Town, South Africa; 6 Department of Psychiatry and Mental Health, University of Cape Town, Cape Town, South Africa; 7 Alcohol, Tobacco & Other Drug Research Unit, South African Medical Research Council, Pretoria, South Africa; 8 Biostatistics Unit, South African Medical Research Council, Cape Town, South Africa; 9 School of Public Health and Family Medicine, University of Cape Town, Cape Town, South Africa; 10 School of Public Health, University of the Witwatersrand, Johannesburg, South Africa; Fordham University, UNITED STATES

## Abstract

**Background:**

Alcohol and other drug use (AOD) and risky sexual behaviours remain high among adolescents in South Africa and globally. Religiosity influences, mitigates and provides resilience against engaging in risky behaviours among young people but few South African studies have explored potential associations between religiosity, AOD use and risky sex. We report the prevalence of religiosity and association between religiosity and AOD use and risky sexual behaviours among learners in the Western Cape Province, South Africa.

**Methods:**

Between May and August 2011, a cross sectional survey was conducted among 20 227 learners from 240 public schools randomly selected through a stratified multistage sampling design to determine the prevalence of AOD use and sexual risk behaviours. We performed univariate and multivariate logistic regression analyses to assess the association between religiosity, AOD use and risky sexual behaviours.

**Results:**

The learners were aged 10–23 years. Almost three quarters (74%) of learners reported high religiosity (defined as attending religious services or activities at least 1–2 times a month). More female than male learners had high religiosity. The prevalence of past 30 day reported alcohol, tobacco and cannabis use was 23%, 19% and 8% respectively. Compared to learners with low religiosity, those with high religiosity were less likely to engage in AOD use: specifically alcohol use, (AOR = 0.86, 95%CI: 0.76–0.97), tobacco use (AOR = 0.76, 95%CI: 0.67–0.87), cannabis use (AOR = 0.57, 95%CI: 0.48–0.68) in the last 30 days. They were also less likely to engage in risky sexual behaviours (AOR = 0.90, 95%CI: 0.81–0.99).

**Conclusion:**

Religiosity was associated with lower odds of reported AOD use and risky sexual behaviours among learners in the Western Cape. This calls for further exploration on how to incorporate religiosity into AOD use and risky sexual behaviour interventions.

## Introduction

Adolescence is viewed as a period of experimentation and heightened risk of engaging in risky behaviours [1–4]. In this period, the most common risk behaviours are alcohol and other drug (AOD) use and risky sexual behaviours that often persist into adulthood [5, 6].

World Health Organisation and United Nations Office on Drugs and Crime reports show that AOD use and risky sexual behaviours continue to be significant public health problems [7–10]. Similar findings are reported by studies carried out in sub-Saharan Africa, including a recent systematic review on alcohol use in eastern Africa, the South African national youth risk behaviour survey, and a large survey on alcohol and other drug use among grade 8–10 learners in Western Cape Province, South Africa. These studies indicate that AOD and risky sexual behaviours are more common among male than female adolescents [11–14]. Since AOD use and risky sexual behaviours usually begin at a young age, potential interventions to address these issues should target adolescents and young people [15, 16].

To develop effective preventative interventions for AOD use and risky sexual behaviours among young people, it is important to identify individual and structural factors that prevent adolescents from initiating AOD use and engaging in risky sexual behaviours. Drug use in South Africa is linked to apartheid laws and the slow socio-economic transformation and equal distribution of wealth proposed in post-apartheid economic policies [17, 18]. Where apartheid legalised racial segregation, failure of economic policies in alleviating poverty among the majority of South African society, has facilitated segregation in the form of class grouping [19]. Affluent individuals tend to live in affluent areas, predominantly those previously demarcated for white people, which tend to be pricier but in turn more secure, have better health care, education, and access to information. Those who continue to live in the areas previously demarcated for black people often do so because of lack of economic power to live elsewhere [19]. These areas tend to have high rates of crime and violence, a legacy of apartheid where law enforcement was primarily used to control black people at the neglect of black-on-black crimes and drug trade and use [20]. These stressful social realities enable a fertile environment of supply and demand for drug trade, with people potentially engaging in drug trade to gain an income in an otherwise limiting economy, while others seek an escape from their everyday reality [17]. In addition, a large part of South Africa is increasingly urbanised, a process that is linked to a decline in traditional social relationships and forms of family structure [17]. Urbanisation is associated with higher rates of unemployment [17] which often leads to high levels of psycho-social distress among parents, making these parents more likely to engage in inconsistent, distant, and harsh parenting [21]. Furthermore, informal employment is also common in urbanised South Africa, which often translates to suboptimal parental supervision of children due to jobs that are physically and time demanding [20].

One of the potentially important modifiers of risky behaviours is religiosity, which continues to be highly prevalent in South Africa despite rapid social change [22]. Religiosity played an important role in the lives of South Africans during the Apartheid period where churches and other places of worship provided people with an opportunity and place for both worship and for meeting to address the political challenges of the era [23]. During the post-Apartheid era, religiosity and spirituality have continued to provide solace and meaning to vast proportions of the population, and particularly, members of disenfranchised communities which face high levels of unemployment and poverty and other social stressors [24].

Religiosity has been widely reported to have a potential for influencing, mitigating, or enhancing resilience with respect to harmful behaviours, thereby promoting good health and general wellbeing among adolescents, young people and adults [25–36]. For example, this literature has identified negative associations between religiosity and sexual risk behaviour, physical inactivity, and poor oral health. Furthermore, it shows that religiosity may be associated with enhanced coping in the face of chronic health conditions. Two systematic reviews reported differentially stronger effects of religiosity and health outcomes among males and older adolescents in comparison to females and younger adolescents [27, 34]. One proposed mechanisms through which religiosity is thought to influence positive health outcomes or behaviours is by imparting meaning, purpose in life, and peace of mind to individuals. Some commentators have argued that religiosity is associated with benefit finding, which refers to the phenomenon of finding positive meaning in negative events [37]. In the face of stressful or traumatic events, those who are higher on religiosity may cope better and be less likely to resort to the use of alcohol or other drugs as coping mechanisms [37]. Furthermore, religiosity also involves social interaction and cohesion within a social (religious) group where the social norms typically discourage engaging in behaviours that may be harmful or risky for health (such as avoiding drugs and alcohol) or may be considered to be morally unacceptable, such as sex before marriage [31, 38, 39]. Consistent with social identity theory, both adolescents and adults with high levels of religiosity (or a high sense of religious social identity) are likely to engage in the behaviours that are prescribed by the group, as they are likely to internalise group norms, and are inclined to want to avoid the risk of social exclusion [40]. Indeed, just as other forms of strong social identity (such as ethnic identity) provide young people with a sense of purpose and belonging, and may be protective against risk behaviours, other forms of prosocial engagements, such as social activism and involvement in political movements may also afford them with a strong sense of belonging to a group and thereby be protective against engagement in risk behaviours [40].

Being religious is not synonymous with having high religiosity. Being a religious person could mean one believes in or belongs to a certain religious faith, while religiosity refers to how frequently one attends organized and non-organized religious activities, worship services, how often one reads religious books or other reading materials, and other domains such as attitude towards religion and religious practices irrespective of the faith being followed [37]. In most studies, whether using a scale or a single item, religiosity has been assessed as the frequency of attending religious activities [32, 41–43].

In South Africa, a few studies carried out among adolescents, university students and adults have examined religiosity. These studies have found that higher religiosity (defined as frequency of attending or participating in religious events/activities) was inversely associated with harmful behaviours or poor health outcomes [32, 44–47]. For example, a small study (n = 90) among adolescents in the Western Cape Province reported on religiosity and drunkenness [48], but no large representative studies, to our knowledge, have reported on the prevalence of religiosity among learners in South Africa or explored associations between religiosity, AOD use and risky sexual behaviours. This study addresses this gap by reporting on the prevalence of religiosity among learners in Western Cape Province, South Africa and its association with AOD use and risky sexual behaviours.

## Methods

### Study setting and population

Between May and August 2011, a cross-sectional survey to determine the prevalence of AOD use and associated factors was carried out among 20 227 young people from 240 public schools in the Western Cape province of South Africa [13]. At around the time of survey, the province had a population of 5.8 million people and an unemployment rate of between 21.6% and 29.3%[49]. A total of 42.5% of the population had at least a Grade 12 education level[49]. The residents of the province consisted of people of various “racial groups”, and the most commonly spoken languages were Afrikaans (49.7%), isiXhosa (24.7%) and English (20.2%). The province has amongst the highest rates of crime and violence, including drug-related crime [50], and a substantial problem of violence and gang-related crime. In addition, the province has high rates of substance use, including alcohol, tobacco and cannabis use [51]. However, it has amongst the lowest rates of sexual risk behaviour and HIV compared to other provinces in South Africa [52].

### Sampling strategy

The survey applied a stratified 2-level cluster sample design. The 49 circuits in the Western Cape were stratified as high, moderate or low risk for adolescent alcohol and drug use using information from police records (total number of drug related crimes reported over 12 months at nearest police station to the school; <200 (0 points), 200–300 (1 point), 500–1000 (2 points) and>1000 (3 points)and information from the Planning and Implementation Management Support -PIMS (decline in matric pass rate of>10% over three years, average pass rate <70% over the three years; 1 point for a school meeting a criteria), and social development offices (school on unsafe school list of department: 1 point) in each circuit. This information was scored over each circuit and the total score divided by number of schools. Circuits with an adjusted score of >3 was classified as high risk (n = 19), score of between 2–3 as moderate risk (n = 15) and <2 as low risk (n = 15). The low risk circuits were pooled into five regional strata and the moderate risk circuits into seven regional strata to ensure regional representation; the high-risk circuits were not pooled, realizing a total of 31 strata. The primary sampling unit within each stratum was a school. Ten schools from each stratum were randomly sampled proportional to the size of the school. In each school, one class in each of three grades (8, 9, and 10) was randomly selected. All learners in the sampled class had an opportunity to participate in the survey.

### Data collection and ethical considerations

#### Study instrument

Learners completed a self-administered questionnaire that was available in the three dominant languages spoken in the Western Cape: English, Afrikaans and isiXhosa. The survey instrument included measures used in previous school surveys on AOD use [53]. Specifically, the questionnaire collected information on socio-demographic characteristics of participants, their school experiences, and home circumstances; lifetime, past year, and last 30 days alcohol, tobacco, and other drug use; treatment for AOD problems; aggressive behaviour, mental health, sexual behaviour and delinquency, community crime and services perceived to be available for youth.

#### Survey administration and ethical considerations

Passive informed consent was obtained from parents/guardians, written informed consent from learners above 18 years as well as written informed assent from those learners whose parents had provided consent for their participation in the study. A trained field team administered the survey. Learners whose caregivers had refused permission for their child to participate and those who did not wish to participate remained in the class during data collection but did not complete the questionnaires. Questionnaire administration took place in the classroom setting with learners completing them individually, overseen by the field team. No teachers or other school personnel were present or permitted to enter the venue while learners were completing the questionnaires. After each learner completed the questionnaire, s/he placed the completed questionnaire in an envelope and placed the sealed envelope in a sealed box. Every learner was given a referral card containing contact details for appropriate organisations dedicated to assisting young people with AOD use, family and other problems. The participants did not receive any reimbursement or compensation for participating in the study. Ethics approval was obtained from the Faculty of Health Sciences Research Ethics Committee at the University of Cape Town (HREC REF: 006/2011). Permission to conduct the survey was obtained from the Western Cape Department of Education.

### Study variables

The outcomes of interest for this paper were self-reported AOD use in the last 30 days (alcohol, tobacco, cannabis) and risky sexual behaviour (defined as reporting at least one of eight possible risky sexual behaviours). The eight sexual behaviour questions were “Did you have sex before your 15th birthday?”, “Have you ever been high on drugs or alcohol when you had sex with someone?”, “Have you had sex with two or more people in the past 3 months?” “Have you ever had anal sex (this means when the penis enters the anus)?” And the following additional questions, “Have you ever been sexually involved with someone who is more than 5 years older than you?”, “Have you ever thought you or your partner might be pregnant?”, “Have you traded sex for money/drugs/alcohol in the past 12 months?)”.

The primary exposure of interest for this paper was religiosity. Participants were classified as having high religiosity if they reported attending a religious service or activity “1–2 times a month or weekly or more”, and low religiosity if they reported “never or rarely” attending a religious service or activity. Other exposures included the mental health status and aggressive behaviour assessed by the Problem Oriented Screening Instrument for Teenagers (POSIT), which is a screening tool validated for use among learners in South Africa [54], academic factors (repeated a grade, ever expelled from school and considering quitting the school), and witnessed a crime. The POSIT scores for mental health were categorized as low risk (1–4), medium risk (5–10) and high risk (≥11). The POSIT scores for aggressive behaviours were categorized as low risk (1–2), medium risk (3–9) and high risk (≥10). The variable “witnessed a crime” was categorised as, “never witnessed a crime event”, “ever witnessed 1 or 2 events”, and “ever witnessed at least 3 events”. The crime events enquired about whether learners had seen someone selling drugs, using drugs, getting shot with a gun, being beaten, getting stabbed, and being forced to have sex when they did not want to.

The demographic variables included in the analyses were sex, school grade, age (categorized as 10–14, 15–17, and 18–23 years), mother’s education (categorized as no formal or less than primary and primary education, secondary education, and college or university education), whether the learner reported living with at least one parent, population group, household financial circumstances and learners’ race. Given the South African context, the participants were asked to self-identify as “White”, “Black”, “Coloured” (of mixed race ancestry) and “Indian.” These refer to demographic markers that are used to monitor socio-economic disparities and do not signify inherent characteristics.

### Data analysis

We used Stata version 14.1 for data analysis. The survey analysis platform in Stata was utilized to account for sampling weight, stratification and clustering. We computed the final study sampling weight using the total number of learners in a school, as provided by the Department of Education for 2010 as a proxy for the total number of learners in grade 8–10 in a school (school level weight) and the probability of sampling a single class within a grade (grade level weight).

The prevalence of reported tobacco, alcohol and cannabis use in the past 30 days, binge drinking in the last two weeks and engagement in at least one risky sexual behaviour was computed. These findings are presented as proportions (%) and 95% CI (Confidence Intervals).

We performed univariate logistic regression to assess the association and the direction of the association of the AOD (alcohol use in the last 30 days, binge drinking in the last 2 weeks, tobacco use in the last 30 days, cannabis use in the last 30 days) and risky sexual behaviours variables with religiosity and other identified exposures. The other identified exposure variables included demographic variables (age, sex, school grade, household financial circumstances), academic factors, and witnessing a crime, mental health and aggressive behaviour scores.

We fitted four multivariate logistic regression models with each of the AOD use variables and risky sexual behaviours. An a priori decision was made that exposure to crime, sex, household income, and school grade are confounders and therefore entered in the multivariate logistic regressions model regardless of the p value [11, 48]. Any other variable that had a p value of ≤0.20 was included in the multivariate logistic regression model. Univariate associations are reported as crude Odds Ratios (OR) and 95% CI and p values, and multivariate associations as adjusted Odds Ratio (AOR) and 95% CI and p values.

## Results

### General characteristics of the study population

We present the findings of 20227 grade 8–10 learners in Western Cape, South Africa who participated in the survey. Overall, the refusal rate was 5% and it was similar across all strata. Table 1 shows that the majority (67%) of the learners were in the 15–17 years age band. Most of the learners identified themselves as Coloured (of mixed race ancestry) (56%) and black African (33%). A total of 58% of learners were living in a household that could afford important, luxury and extra goods. Amongst all learners, 88% were living with either or both parents. A quarter of the learners had repeated a grade after failing an exam and 23% had considered quitting school. Most of learners (74%) reported high religiosity, with more females than males having high religiosity (76% vs 70%, p <0.001). Two thirds of the learners had witnessed one or more crime event in the last 12 months and this was not significantly different between females and males. More than half of the learners were at medium to high risk for mental health problems (55%) and aggressive behaviours (63%) respectively. The prevalence of reported alcohol use in the last 30 days was 23% (95%CI: 21.7–24.1), and that of binge drinking in the past two weeks was 14% (95%CI: 13.6–15.4). Tobacco and cannabis use were fairly common with 19% (95%CI: 17.9–20.2) and 8% (95%CI: 7.3–8.7) of learners reporting last 30 days tobacco and cannabis use respectively. Among the learners, 28% (95%CI: 26.2–29.5) reported ever having engaged in at least one risky sexual behaviour.

**Table 1:** The characteristics of grade 8–10 learners in Western Cape Province, South Africa, 2011.

Characteristic	categories	Overall			Female students			Male students		
		n	%	95% CI	n	%	95% CI	n	%	95% CI
Age	10–14 years	4 934	23.8	[21.2,26.7]	3 051	25.8	[22.8,29.0]	1 883	21.2	[18.6,24.2]
	15–17 years	13 200	67.6	[65.0,70.1]	7 417	67.1	[64.1,70.0]	5 783	68.3	[65.5,70.9]
	18–23 years	1 569	8.6	[7.5,9.8]	739	7.2	[6.0,8.5]	830	10.5	[9.1,12.1]
School grade	8	7 037	31.9	[28.3,35.8]	4 024	32.0	[28.2,36.1]	3 013	31.7	[27.9,35.8]
	9	6 693	34.5	[30.7,38.5]	3 604	32.7	[28.9,36.9]	3 089	36.9	[32.7,41.2]
	10	6 365	33.6	[29.8,37.6]	3 779	35.2	[31.1,39.6]	2 586	31.4	[27.6,35.5]
Religiosity	Low religiosity	5 022	26.1	[25.1,27.2]	2 618	23.9	[22.8,25.1]	2 404	29.1	[27.3,31.0]
	High religiosity	14 534	73.9	[72.8,74.9]	8 480	76.1	[74.9,77.2]	6 054	70.9	[69.0,72.7]
Population group	Black African	6 764	32.9	[29.4,36.6]	3 868	33.2	[29.4,37.3]	2 896	32.5	[29.0,36.3]
	Coloured	10 943	55.7	[52.2,59.3]	6 261	56.2	[52.4,59.9]	4 682	55.1	[51.1,59.1]
	Indian and other	322	1.7	[1.4,2.1]	177	1.7	[1.3,2.3]	145	1.7	[1.3,2.1]
	White	1 877	9.6	[7.5,12.2]	994	8.8	[6.6,11.8]	883	10.7	[7.6,14.7]
Household income	No enough money for food	1 287	7.1	[6.2,8.0]	692	6.7	[5.7,7.9]	595	7.5	[6.6,8.6]
	Enough money for food but not for other basic items like clothes	2 115	11.2	[10.3,12.2]	1 276	11.9	[10.7,13.1]	839	10.4	[9.3,11.6]
	Enough money for food and clothes but short of many other things	4 614	23.7	[22.5,24.9]	2 631	23.5	[22.0,25.0]	1 983	23.9	[22.2,25.8]
	Enough money for important and luxury and extra goods	10 967	58.0	[55.7,60.3]	6 259	57.9	[55.1,60.7]	4 708	58.2	[55.1,61.1]
Repeated a grade in school due to failure	No	14 934	75.1	[73.3,76.7]	8 945	79.3	[77.4,81.0]	5 989	69.5	[66.9,72.0]
	Yes	4 992	24.9	[23.3,26.7]	2 375	20.7	[19.0,22.6]	2 617	30.5	[28.0,33.1]
Considered quitting school	No	15 526	77.4	[76.2,78.6]	8 871	77.9	[76.3,79.4]	6 655	76.8	[75.4,78.2]
	Yes	4 357	22.6	[21.4,23.8]	2 429	22.1	[20.6,23.7]	1 928	23.2	[21.8,24.6]
Ever expelled from school	No	18 345	92.8	[92.1,93.5]	10 686	95.4	[94.8,96.0]	7 659	89.3	[88.0,90.4]
	Yes	1 400	7.2	[6.5,7.9]	512	4.6	[4.0,5.2]	888	10.7	[9.6,12.0]
Parenting	Do not live with Father or Mother	2 148	12.0	[10.6,13.5]	1 199	12.1	[10.5,13.9]	949	11.9	[10.4,13.6]
	Live with either Father or Mother	15 493	88.0	[86.5,89.4]	8 687	87.9	[86.1,89.5]	6 806	88.1	[86.4,89.6]
Mother's education	Primary or less	2 510	13.4	[12.6,14.3]	1 456	13.6	[12.6,14.7]	1 054	13.2	[12.0,14.4]
	Secondary	8 083	42.1	[40.9,43.4]	4 665	42.7	[41.1,44.3]	3 418	44.4	[39.6,43.2]
	College or university	4 526	24.0	[22.0,26.0]	2 477	23.3	[20.8,26.0]	2 049	24.9	[22.3,27.7]
	Do not know	3 911	20.5	[19.6,21.4]	2 222	20.4	[19.2,21.6]	1 689	20.6	[19.5,21.8]
Tobacco use in the last month	No	16 078	81.0	[79.8,82.1]	9 325	82.9	[81.6,84.2]	6 753	78.4	[76.8,79.9]
	Yes	3 838	19.0	[17.9,20.2]	1 992	17.1	[15.8,18.4]	1 846	21.6	[20.1,23.2]
Alcohol use in the last month	No	15 449	77.1	[75.9,78.3]	8 854	77.7	[76.3,79.0]	6 595	76.3	[74.7,77.9]
	yes	4 370	22.9	[21.7,24.1]	2 392	22.3	[21.0,23.7]	1 978	23.7	[22.1,25.3]
Binge drinking in the past two weeks (had 5 or more drink once in a short time)	No	17 041	85.6	[84.6,86.4]	9 851	87.2	[86.2,88.2]	7 190	83.3	[82.1,84.6]
	Yes	2 778	14.4	[13.6,15.4]	1 398	12.8	[11.8,13.8]	1 380	16.7	[15.4,17.9]
Dagga(Cannabis) use in the last month	No	18 320	92.0	[91.3,92.7]	10 611	94.1	[93.4,94.8]	7 709	89.1	[87.9,90.2]
	Yes	1 610	8.0	[7.3,8.7]	703	5.9	[5.2,6.6]	907	10.9	[9.8,12.1]
Mental status	Low risk	8 970	44.8	[43.6,46.0]	4 248	37.6	[36.0,39.3]	4 722	54.3	[52.7,56.0]
	Medium risk	8 188	40.6	[39.7,41.5]	5 089	44.2	[43.0,45.5]	3 099	35.8	[34.4,37.3]
	High risk	2 937	14.6	[13.8,15.4]	2 070	18.2	[17.1,19.3]	867	9.8	[9.1,10.5]
Aggressive behaviour	Low risk	6 196	30.6	[29.3,31.9]	3 396	29.9	[28.4,31.4]	2 800	31.5	[29.9,33.2]
	Medium risk	12 577	62.7	[61.5,63.8]	7 311	63.8	[62.4,65.1]	5 266	61.2	[59.6,62.8]
	High risk	1 322	6.7	[6.2,7.3]	700	6.3	[5.7,7.0]	622	7.2	[6.5,8.1]
Risky sexual behaviours	No risky behaviour reported	14 539	72.2	[70.5,73.8]	8 879	77.4	[75.5,79.2]	5 660	65.2	[63.1,67.3]
	At least one risky behaviour reported	5 556	27.8	[26.2,29.5]	2 528	22.6	[20.8,24.5]	2 028	34.8	[32.7,36.9]
Witnessing a crime event	Not witnessed any crime event	6 318	32.3	[30.9,33.8]	3 352	30.0	[28.0,32.1]	2 966	35.4	[33.8,37.1]
	Witnessed 1–2 crime events	5 684	28.2	[27.2,29.1]	3 512	30.8	[29.6,32.0]	2 172	24.6	[23.2,26.1]
	Witnessed more than 3 crime events	8 093	39.5	[37.7,41.3]	4 543	39.2	[36.9,41.5]	3 550	40.0	[37.7,42.3]

### Religiosity and alcohol use in the last 30 days

In multivariate analyses, learners with high religiosity were significantly less likely to report using alcohol in the last 30 days (AOR = 0.86, 95%CI: 0.76–0.97). Alcohol use in the last 30 days was also positively associated with belonging in the age band 15–17 years, being in a higher school grade than grade 8 (9 or 10), and experiencing adverse academic events. In addition, participants who reported having enough money for important and luxury and extra goods, being White, and having a medium or high risk POSIT score for mental health and aggressive behaviours, were more likely to report alcohol use in the last 30 days than those below the median scores (Table 2).

**Table 2:** The association of alcohol use in the last 30 days with religiosity among grade 8–10 learners in Western Cape Province, South Africa, 2011.

Characteristic	categories	Reported alcohol use		Crude estimates			Adjusted estimates^3^		
		N^1^	%^2^	OR	95% CI	p value	OR	95% CI	p value
Religiosity	Low religiosity	1 250	26.1	1			1		
	**High religiosity**	**3 076**	**22.2**	**0.81**	**[0.73,0.89]**	**<0.001**	**0.86**	**[0.76,0.97]**	**0.014**
Age						***<0*.*001***			*0*,*059*
	10–14 years	584	12.6	1			1		
	15–17 years	3 273	25.7	**2.40**	**[2.06,2.77]**	**<0.001**	**1.30**	**[1.05,1.62]**	**0.017**
	**18–23 years**	**465**	**30.1**	**2.98**	**[2.13, 2.84]**	**<0.001**	**1.37**	**[0.99,1.91]**	**0.060**
School grade						***<0*.*001***			***<0*.*001***
	8	868	12.9	1			1		
	**9**	**1 539**	**24.0**	**2.12**	**[1.82, 2.48]**	**<0.001**	**1.66**	**[1.36,2.04]**	**<0.001**
	**10**	**1 992**	**31.3**	**3.07**	**[2.63, 3.57]**	**<0.001**	**2.44**	**[1.95, 3.04]**	**<0.001**
Sex	Female	2 392	22.3	1			1		
	Male	1 978	23.7	1.08	[0.98, 1.20]	0.123	1.00	[0.89,1.12]	0.998
Population group						***<0*.*001***			***<0*.*001***
	Black African	1 188	18,7	1			1		
	Coloured	2 561	24,1	1.39	[1.21, 1.58]	<0.001	1.11	[0.97,1.28]	0.118
	Indian and other	65	20,9	1.15	[0.81, 1.64]	0.417	0.84	[0.56,1.26]	0.398
	**White**	**563**	**31,6**	**2.01**	**[1.60, 2.53]**	**<0.001**	**1.92**	**[1.54,2.41]**	**<0.001**
Household income						***<0*.*001***			***<0*.*001***
	No enough money for food	219	18.2	1			1		
	Enough money for food but not for other basic items like clothes	394	19.3	1.08	[0.86, 1.35]	0.533	0.99	[0.74,1.34]	0.972
	Enough money for food and clothes but short of many other things	940	21.2	1.21	[0.99, 1.47]	0.066	1.17	[0.90,1.52]	0.241
	**Enough money for important and luxury and extra goods**	**2 651**	**25.2**	**1.51**	**[1.25, 1.83]**	**<0.001**	**1.40**	**[1.10,1.78]**	**<0.001**
Repeated a grade in school due to failure	No	3 004	21.2	1			1		
	**Yes**	**1 370**	**28.3**	**1.46**	**[1.31, 1.62]**	**<0.001**	**1.27**	**[1.10,1.46]**	**0,001**
Considered quitting school	No	2 778	18.8	1			1		
	**Yes**	**1 587**	**37.2**	**2.55**	**[2.30, 2.82]**	**<0.001**	**1.58**	**[1.38,1.80]**	**<0.001**
Ever expelled from school	No	3 797	21.7	1			1		
	**Yes**	**545**	**39.7**	**2.38**	**[2.04, 2.76]**	**<0.001**	**1.74**	**[1.45,2.09]**	**<0.001**
Parenting	Do not live with Father or Mother	412	20.0	1			1		
	Live with either Father or Mother	3 553	24.1	1.27	[1.10, 1.47]	0.001	0.92	[0.77,1.10]	0.334
Mother's education						***<0*.*001***			***0*,*001***
	Primary or less	537	22.6	1			1		
	Secondary	1 799	23.1	1.03	[0.90, 1.17]	0.690	0.99	[0.85,1.15]	0.865
	**College or university**	**1 131**	**26.3**	**1.22**	**[1.05, 1.42]**	**0.011**	**1.20**	**[1.01,1.42]**	**0.039**
	Do not know	727	19.5	0.83	[0.71, 0.96]	0.014	0.90	[0.74,1.08]	0.263
Mental status						***<0*.*001***			***<0*.*001***
	Low risk	1 385	16.0	1			1		
	**Medium Risk**	**2 034**	**26.1**	**1.84**	**[1.69, 2.01]**	**<0.001**	**1.27**	**[1.13,1.41]**	**<0.001**
	**High risk**	**980**	**35.1**	**2.83**	**[2.51,3.20]**	**<0.001**	**1.36**	**[1.16,1.60]**	**<0.001**
Aggressive behaviour						***<0*.*001***			***<0*.*001***
	Low risk	638	11.0	1			1		
	**Medium risk**	**3 066**	**25.3**	**2.73**	**[2.44, 3.06]**	**<0.001**	**2.02**	**[1.78,2.31]**	**<0.001**
	**High risk**	**695**	**54.4**	**9.63**	**[8.15,11.37]**	**<0.001**	**5.42**	**[4.36,6.72]**	**<0.001**
Witnessing a crime event						***<0*.*001***			***<0*.*001***
	Not witnessed any crime event	1 039	17.7	1			1		
	Witnessed 1–2 crime events	1 076	20.4	1.20	[1.04, 1.37]	0.011	1.06	[0.91,1.22]	0.458
	**Witnessed more than 3 crime events**	2 284	29.0	1.90	[1.69, 2.13]	**<0.001**	**1.32**	**[1.15,1.52]**	**<0.001**

^1^ Learner’s reported Alcohol use in the last 30 days

^2^ Proportion of learners reporting alcohol use across the exposure categories

^3^ Adjusted for all variables in this table

### Religiosity and tobacco use in the last 30 days

Learners with high religiosity were significantly less likely to report using tobacco in the last 30 days than learners with low religiosity (AOR = 0.76, 95%CI: 0.67–0.87). Tobacco use in the last 30 days was also associated with living with at least one parent, being in a higher grade (9 or 10), race, adverse academic events and being a male learner. Tobacco use was further associated with medium and high risk POSIT scores for mental health and aggressive behaviours and witnessing more than three crime events (Table 3).

**Table 3:** The association of tobacco use in the last 30 days with religiosity among grade 8–10 learners in Western Cape Province, South Africa, 2011.

Characteristic	categories	Reported tobacco use		Crude estimates			Adjusted estimates^3^		
		N^1^	%^2^	OR	95% CI	p value	OR	95% CI	p value
**Religiosity**	Low religiosity	1 193	23.2	1			1		
	**High religiosity**	**2 621**	**17.9**	**0.72**	**[0.66,0.80]**	**<0.001**	**0.76**	**[0.67, 0.87]**	**<0.001**
**Age**						***<0*.*001***			*0*.*460*
	10–14 years	512	11.2	1			1		
	15–17 years	2 854	20.8	2.09	[1.79,2.45]	<0.001	1.03	[0.81,1.32]	0.781
	18–23 years	435	26.7	2.89	[2.35, 3.58]	<0.001	1.20	[0.84, 1.73]	0.313
**School grade**						***<0*.*001***			*<0*.*001*
	8	826	12.3	1			1		
	**9**	**1 370**	**20.5**	**1.83**	**[1.53,2.18]**	**<0.001**	**1.62**	**[1.31,2.01]**	**<0.001**
	**10**	**1 664**	**23.8**	**2.22**	**[1.86,2.66]**	**<0.001**	**2.09**	**[1.69, 2.59]**	**<0.001**
**Sex**	Female	1 992	17.1	1			1		
	**Male**	**1 846**	**21.6**	**1.34**	**[1.21,1.48]**	**<0.001**	**1.19**	**[1.04, 1.36]**	**0.009**
**Population group**						***<0*.*001***			*<0*.*001*
	Black African	645	9.6	1			1		
	**Coloured**	**2 770**	**24.7**	**3.10**	**[2.68,3.58]**	**<0.001**	**2.94**	**[2.44, 3.55]**	**<0.001**
	**Indian**	**66**	**20.0**	**2.36**	**[1.62,3.44]**	**<0.001**	**1.98**	**[1.31, 2.99]**	**0.001**
	**White**	**354**	**18.3**	**2.11**	**[1.69,2.63]**	**<0.001**	**2.57**	**[1.99, 3.32]**	**<0.001**
**Household income**						***0*.*024***			*0*.*904*
	No enough money for food	219	17.0	1			1		
	Enough money for food but not for other basic items like clothes	362	16.7	0.98	[0.79, 1.20]	0.819	0.91	[0.68, 1.22]	0.535
	Enough money for food and clothes but short of many other things	868	18.9	1.14	[0.94, 1.39]	0.184	0.95	[0.72, 1.26]	0.727
	Enough money for important and luxury and extra goods	2 246	20.2	1.24	[1.01,1.53]	0.041	0.97	[0.74,1.27]	0.815
**Repeated a grade in school due to failure**	No	2 405	15.9	1			1		
	**Yes**	**1 430**	**28.5**	**2.11**	**[1.89,2.34]**	**<0.001**	**1.82**	**[1.57, 2.12]**	**<0.001**
**Considered quitting school**	No	2 220	14.1	1			1		
	**Yes**	**1 612**	**36.0**	**3.43**	**[3.07, 3.83]**	**<0.001**	**1.92**	**[1.67, 2.21]**	**<0.001**
**Ever expelled from school**	No	3 231	17.4	1			1		
	**Yes**	**585**	**41.6**	**3.38**	**[2.92,3.91]**	**<0.001**	**2.54**	**[2.14, 3.02]**	**<0.001**
**Parenting**	Do not live with Father or Mother	230	10.2	1			1		
	**Live with either Father or Mother**	**3 200**	**20.5**	**2.28**	**[1.90,2.74]**	**<0.001**	**1.29**	**[1.06, 1.58]**	**0.013**
**Mother's education**						***0*.*174***			*0*.*176*
	Primary or less	482	18.8	1			1		
	**Secondary**	**1 664**	**20.0**	**1.08**	**[0.94, 1.24]**	**0.267**	**1.19**	**[1.00, 1.41]**	**0.055**
	College or university	806	17.9	0.94	[0.79, 1.12]	0.515	1.12	[0.92, 1.36]	0.243
	**Do not know**	**732**	**19.1**	**1.02**	**[0.87, 1.19]**	**0.824**	**1.22**	**[1.00, 1.48]**	**0.049**
**Mental status**						**<0.001**			0.054
	Low risk	1 185	13.2	1			1		
	**Medium risk**	**1 711**	**20.9**	**1.74**	**[1.57, 1.92]**	**<0.001**	**1.11**	**[0.98,1.28]**	**0.102**
	**High risk**	**964**	**31.8**	**3.07**	**[2.72, 3.48]**	**<0.001**	**1.26**	**[1.04,1.52]**	**0.016**
**Aggressive behaviour**						***<0*.*001***			*<0*.*001*
	Low risk	555	8.8	1			1		
	**Medium risk**	**2 648**	**20.8**	**2.73**	**[2.39, 3.10]**	**<0.001**	**1.92**	**[1.65, 2.23]**	**<0.001**
	**High risk**	**657**	**48.9**	**9.92**	**[8.30,11.85]**	**<0.001**	**4.61**	**[3.61,5.89]**	**<0.001**
**Witnessing a crime event**						***<0*.*001***			*<0*.*001*
	Not witnessed any crime event	912	14.6	1			1		
	Witnessed 1–2 crime events	862	15.4	1.07	[0.93,1.23]	0.360	0.96	[0.83, 1.12]	0.716
** **	**Witnessed more than 3 crime events**	**2 086**	**25.2**	**1.98**	**[1.73,2.26]**	**<0.001**	**1.43**	**[1.23, 1.66]**	**<0.001**

^1^ Learner’s reported tobacco use in the last 30 days

^2^ Proportion of learners reporting tobacco use across the exposure categories

^3^ Adjusted for all variables in the table

### Religiosity and cannabis use in the last 30 days

Compared to learners with low religiosity, learners with high religiosity had diminished odds of reporting cannabis use in the last 30 days (AOR = 0.57, 95%CI: 0.48–0.68). In addition, cannabis use in the last 30 days was associated with witnessing 3 or more crime events, adverse academic events and medium and high risk POSIT scores for mental health and aggressive behaviour. Race, male learners, and belonging to grade 9 or 10 were also significantly associated with reported cannabis use in the last 30 days (Table 4).

**Table 4:** The association of cannabis use in the last 30 days with religiosity among grade 8–10 learners in Western Cape Province, South Africa, 2011.

Characteristic	categories	Reported cannabis use		Crude estimates			Adjusted estimates^3^		
		N^1^	%^2^	OR	95% CI	p value	OR	95% CI	p value
**Religiosity**	Low religiosity	606	11.7	1			1		
	**High religiosity**	**989**	**6.9**	**0.56**	**[0.48,0.64]**	**<0.001**	**0.57**	**[0.48,0.68]**	**<0.001**
**Age**						***<0*.*001***			*0*,*690*
	10–14 years	171	3.9	1			1		
	15–17 years	1 248	9.1	2.45	[1.96,3.05]	<0.001	1.01	[0.72,1.42]	0,958
	18–23 years	170	10.5	2.86	[2.00,3.11]	<0.001	0.88	[0.55, 1.40]	0,596
**School grade**						***<0*.*001***			*<0*.*001*
	8	294	4.3	1			1		
	**9**	**615**	**9.0**	**2.16**	**[1.73, 2.72]**	**<0.001**	**2.06**	**[1.49, 2.86]**	**<0.001**
	**10**	**711**	**10.5**	**2.59**	**[2.09, 3.21]**	**<0.001**	**2.46**	**[1.78, 3.40]**	**<0.001**
**Sex**	Female	703	5.9	1			1		
	**Male**	**907**	**10.9**	**1.96**	**[1.67, 2.30]**	**<0.001**	**1.85**	**[1.51, 2.28]**	**<0.001**
**Population group**						***<0*.*001***			***<0*.*001***
	Black African	348	4.9	1			1		
	**Coloured**	**1 128**	**10.3**	**2.21**	**[1.82, 2.68]**	**<0.001**	**1.92**	**[1.55, 2.37]**	**<0.001**
	Indian	30	9.8	2.08	[1.28, 3.38]	0,003	1.42	[0.75, 2.68]	0.283
	White	105	5.4	1.10	[0.80, 1.52]	0,544	1.14	[0.76, 1.70]	0.534
**Household income**						*0*.*670*			*0*.*684*
	No enough money for food	112	8.3	1			1		
	Enough money for food but not for other basic items like clothes	155	7.2	0.86	[0.60, 1.21]	0.381	0.74	[0.45,1.22]	0.236
	Enough money for food and clothes but short of many other things	370	7.9	0.95	[0.71, 1.29]	0.761	0.79	[0.52,1.21]	0.274
	Enough money for important and luxury and extra goods	900	8.3	1.00	[0.75, 1.33]	0.994	0.79	[0.52, 1.20]	0.260
**Repeated a grade in school due to failure**	No	1 002	6.8	1			1		
	**Yes**	**607**	**11.8**	**1.84**	**[1.58, 2.15]**	**<0.001**	**1.32**	**[1.09, 1.60]**	**0.005**
**Considered quitting school**	No	884	5.7	1			1		
	**Yes**	**725**	**16.0**	**3.14**	**[2.76, 3.56]**	**<0.001**	**1.76**	**[1.51, 2.06]**	**<0.001**
**Ever expelled from school**	No	1 281	7.0	1			1		
	**Yes**	**317**	**22,5**	**3.89**	**[3.20, 4.73]**	**<0.001**	**2.27**	**[1.78, 2.89]**	**<0.001**
**Parenting**	Do not live with Father or Mother	108	5.3	1			1		
	Live with either Father or Mother	1 351	8.6	166	[1.29, 2.14]	<0.001	1.26	[0.94, 1.71]	0.125
**Mother's education**						*0*.*103*			*0*.*830*
	Primary or less	222	8.1	1			1		
	Secondary	714	8.6	1.07	[0.86, 1.32]	0,566	1.10	[0.85, 1.42]	0.471
	College or university	306	7.1	0.87	[0.67, 1.14]	0,320	1.03	[0.75,1.43]	0.834
	Do not know	286	7.5	0.91	[0.72, 1.16]	0,464	1.03	[0.78, 1.37]	0.822
**Mental status**						***<0*.*001***			*0*.*300*
	Low risk	483	5.4	1			1		
	**Medium risk**	**697**	**8.7**	**1.68**	**[1.42, 2.00]**	**<0.001**	1.17	[0.95, 1.45]	0.139
	**High risk**	**440**	**14.3**	**2.95**	**[2.44,3.56]**	**<0.001**	1.21	[0.91, 1.61]	0.191
**Aggressive behaviour**						***<0*.*001***			***<0*.*001***
	Low risk	190	3.3	1			1		
	**Medium risk**	**1 046**	**8.2**	**2.58**	**[2.13, 3.13]**	**<0.001**	**1.88**	**[1.54, 2.29]**	**<0.001**
	**High risk**	**384**	**28.0**	**11.31**	**[8.77,14.58]**	**<0.001**	**5.20**	**[3.91,6.91]**	**<0.001**
**Witnessing a crime event**						***<0*.*001***			***<0*.*001***
	Not witnessed any crime event	314	5.0	1			1		
	Witnessed 1–2 crime events	301	5.5	1.12	[0.82, 1.53]	0.466	1,13	[0.81,1.59]	0.462
** **	**Witnessed more than 3 crime events**	**1 005**	**12.3**	**2.67**	**[2.11, 3.38]**	**<0.001**	**1,87**	**[1.48, 2.37]**	**<0.001**

^1^ Learner’s reported cannabis use in the last 30 days

^2^ Proportion of learners reporting dagga use across exposure categories

^3^ Adjusted for all variables in the table

### Religiosity and risky sexual behaviours

The results of the multivariate analyses, learners with high religiosity were significantly less likely to report sexual risk behaviour (AOR = 0.90, 95%CI: 0.81–0.99) relative to learners with low religiosity. Reporting at least one risky sexual behaviour was significantly associated with medium and high risk POSIT scores for mental health and aggressive behaviours, adverse academic events, and being male and the race of the learner (Table 5).

**Table 5:** The association of risky sexual behaviours with religiosity among grade 8–10 learners in Western Cape Province, South Africa, 2011.

Characteristic	categories	Reported at least 1 risky sexual behaviour		Crude estimates			Adjusted estimates^3^		
		N^1^	%^2^	OR	95% CI	p value	OR	95% CI	p value
**Religiosity**	Low religiosity	1 609	31.8	1			1		
	**High religiosity**	**3 849**	**26.5**	**0.78**	**[0.71, 0.85]**	**<0.001**	**0.90**	**[0.81,0.99]**	**0.049**
**Age**						***<0*.*001***			***<0*.*001***
	10–14 years	684	13.3	1			1		
	**15–17 years**	**3 957**	**29.7**	**2.75**	**[2.41,3.15]**	**<0.001**	**1.59**	**[1.27,2.00]**	**<0.001**
	**18–23 years**	**825**	**52.4**	**7.19**	**[5.98, 8.63]**	**<0.001**	**1.98**	**[1.42,2.77]**	**<0.001**
**School grade**						***<0*.*001***			***<0*.*001***
	8	1 267	16.9	1			1		
	**9**	**1 859**	**27.4**	**1.85**	**[1.60, 2.15]**	**<0.001**	**1.25**	**[1.00, 1.55]**	**0.045**
	**10**	**2 461**	**38.6**	**3.09**	**[2.68, 3.56]**	**<0.001**	**2.01**	**[1.61, 2.52]**	**<0.001**
**Sex**	Female	2 528	22.6	1			1		
	**Male**	**3 028**	**34.8**	**1.83**	**[1.65, 2.03]**	**<0.001**	**2.21**	**[1.97, 2.48]**	**<0.001**
**Population group**						***<0*.*001***			***<0*.*001***
	Black African	2 419	36.9	1			1		
	**Coloured**	**2 743**	**24.6**	**0.56**	**[0.49, 0.64]**	**<0.001**	**0.38**	**[0.33, 0.45]**	**<0.001**
	**Indian and Other**	**81**	**23.7**	**0.53**	**[0.37, 0.76]**	**0.001**	**0.37**	**[0.24, 0.58]**	**<0.001**
	**White**	**312**	**17.0**	**0.35**	**[0.28, 0.44]**	**<0.001**	**0.34**	**[0.27, 0.44]**	**<0.001**
**Household income**						***<0*.*001***			***0*.*017***
	No enough money for food	450	36.9	1			1		
	Enough money for food but not for other basic items like clothes	731	35.6	0.95	[0.78, 1.15]	0.596	1.13	[0.86, 1.49]	0.362
	Enough money for food and clothes but short of many other things	1 447	31.7	0.80	[0.67,0.95]	0.010	1.07	[0.84, 1.36]	0.591
	Enough money for important and luxury and extra goods	2 665	23.7	0.53	[0.44, 0.63]	<0.001	0.91	[0.72, 1.15]	0.429
**Repeated a grade in school due to failure**	No	3 402	22.7	1			1		
	**Yes**	**2 146**	**43.4**	**2.60**	**[2.36, 2.87]**	**<0.001**	**1.71**	**[1.52, 1.93]**	**<0.001**
**Considered quitting school**	No	3 693	23.7	1			1		
	**Yes**	**1 838**	**41.9**	**2.32**	**[2.10, 2.56]**	**<0.001**	**1.43**	**[1.27, 1.60]**	**<0.001**
**Ever expelled from school**	No	4 772	26.1	1			1		
	**Yes**	**700**	**49.1**	**2.74**	**[2.34, 3.20]**	**<0.001**	**1.59**	**[1.33, 1.90]**	**<0.001**
**Parenting**	Do not live with Father or Mother	656	31.9	1			1		
	Live with either Father or Mother	4 158	26.5	0.77	[0.66, 0.90]	0,001	1.11	[0.94, 1.32]	0.208
**Mother's education**						***<0*.*001***			***0*.*191***
	Primary or less	812	33.6	1			1		
	Secondary	2 333	29.1	0.81	[0.72, 0.91]	<0.001	0.89	[0.77,1.04]	0.139
	College or university	1 145	24.4	0.64	**[0.54, 0.76]**	**<0.001**	**0.82**	**[0.69,0.98]**	**0.030**
	Do not know	986	25.2	0.67	[0.58, 0.76]	<0.001	0.87	[0.73,1.04]	0.129
**Mental status**						***<0*.*001***			***<0*.*001***
	Low risk	1 799	20.1	1			1		
	**Medium risk**	**2 538**	**31.4**	**1.82**	**[1.67, 1.99]**	**<0.001**	**1.54**	**[1.37, 1.73]**	**<0.001**
	**High risk**	**1 250**	**41.7**	**2.85**	**[2.55,3.18]**	**<0.001**	**2.08**	**[1.77,2.44]**	**<0.001**
**Aggressive behaviour**						***<0*.*001***			***<0*.*001***
	Low risk	832	13.9	1			1		
	**Medium risk**	**4 002**	**31.6**	**2.87**	**[2.58,3.20]**	**<0.001**	**2.61**	**[2.27, 3.01]**	**<0.001**
	**High risk**	**753**	**55.9**	**7.88**	**[6.60,9.41]**	**<0.001**	**5.87**	**[4.69,7.33]**	**<0.001**
**Witnessing a crime event**						***<0*.*001***			***<0*.*001***
	Not witnessed any crime event	1 204	18.9	1			1		
	**Witnessed 1–2 crime events**	**1 371**	**24.4**	**1.38**	**[1.24, 1.54]**	**<0.001**	**1.54**	**[1.34, 1.78]**	**<0.001**
** **	**Witnessed more than 3 crime events**	**3 012**	**37.6**	**2.58**	**[2.29, 2.91]**	**<0.001**	**2.21**	**[1.91, 2.56]**	**<0.001**

^1^ Learner’s reported at least one risky sexual behaviour

^2^ Proportion of learners reporting risk sexual behaviour across exposure categories

^3^ Adjusted for all variables in the table

## Discussion

To the best of our knowledge, this is the first large study in South Africa to report on the prevalence of high religiosity and the association of high religiosity with AOD use and risky sexual behaviours. Most learners reported high religiosity (i.e. attendance of religious activities at least once-twice per month) and this was higher among female learners than among male learners. The results also indicate high levels of AOD use and risky sexual behaviours. These results are consistent with the literature on substance use and sexual risk behaviour in sub-Saharan Africa [11, 12, 14–16]. Our findings show that learners with high religiosity had significantly reduced odds of AOD use in the last 30 days, and risky sexual behaviours. Furthermore, similar to other studies among adolescents and young people in South Africa and other parts of Africa, poor academic performance, higher scores of mental health status and aggressive behaviours, being male and having witnessed a crime were associated with AOD use [12, 54–57].

Our findings further confirm the link between higher religiosity and positive health behaviours, which is in agreement with previous studies conducted among young adults and adults in South Africa [46, 47, 58], and other countries [25, 26, 28, 29, 31–34, 43]. These studies reported that higher religiosity has inverse association with risky behaviours and poor health outcomes, suggesting that religiosity may be a potential avenue for inclusion into AOD use and risky sexual behaviour interventions among adolescents and young people. Even though religiosity has had positive effects on health outcomes, using faith-based platforms for public health interventions is welcomed but should be evidence-based [59]. Nevertheless, health intervention providers for young people could explore and strengthen religiosity among those reporting to be religious but with low religiosity. For example, in the USA, Griffith et al (2010) implemented an HIV intervention, bridging faith-based platforms and the public health communities successfully [60].

Our study included a large and representative sample of grade 8–10 learners in Western Cape Province, however, the findings should be interpreted in light of the limitations highlighted below. First, the study design was cross-sectional and therefore it is not possible to ascertain the temporal relationship between religiosity and AOD use and risky sexual behaviours. Second, learners self-reported their religiosity, AOD use, and risky sexual behaviours and therefore there was a risk of social desirable responses that would lead to underestimation of the magnitude of AOD use and risky sexual behaviours, thereby biasing our results towards the null. We attempted to reduce the social desirability responses by using self-administered questionnaires and ensuring anonymity. Third, we acknowledge the potential of misclassification due to poor recall, especially regarding the questions on risky sexual behaviours, such as age at sexual debut. Fourth, this being a secondary analysis of the behavioural survey data, we could not include prosocial influences such as cultural activities/political movements/ social activism that may provide a space where young people feel a sense of belonging and purpose and could lead to either increased or reduced risk behaviours. Fifth, we used a narrow definition of religiosity, which did not include elements of spirituality. Sixth, we did not assess post-traumatic stress disorder which could potentially increase the risky behaviours but we adjusted all the analyses for mental health and aggressive behaviour status. Finally, the findings may only be generalizable to the population of learners in public schools in the Western Cape Province and a similar population in South Africa.

This study’s findings call for further exploration of how religious practice could serve as a platform for AOD use and risky sexual behaviours interventions. As a starting point, one could borrow a leaf from a faith-based intervention in the USA that encouraged a discussion of health topics among youths during or after the religious services for the AOD use and risky sexual behaviours. Furthermore we could use findings of establishment youth ministry on the influence of youth development in Western Cape, South Africa [22, 60]. Another potential avenue is to include religiosity in AOD use and risky sexual behaviour reduction interventions by promoting higher religiosity among those religious adolescents with low religiosity who are already using AODs. In addition, based on the tenets of social identity theory and from the findings of this paper, we could identify and assess interventions that have included the engagement of adolescents and young people in (secular or religious) prosocial networks, which could give them a stronger sense of belonging and purpose [40]. In addition, since parenting has proven to be an effective way to modify substance use among adolescents and young people, religiosity could be included as part of parenting interventions [61].

## References

[1] Casey B, Caudle K. The Teenage Brain: Self Control. Curr Dir Psychol Sci. 2013;22(2):82–7. 10.1177/0963721413480170 25284961 PMC4182916

[2] Dayan J, Bernard A, Olliac B, Mailhes AS, Kermarrec S. Adolescent brain development, risk-taking and vulnerability to addiction. J Physiol Paris. 2010;104(5):279–86. 10.1016/j.jphysparis.2010.08.007 . 20816768

[3] Furby LB-M, R;. Risk Taking in Adolescence: A Decision-Making Perspective. DEVELOPMENTAL REVIEW. 1992;12:1–44.

[4] Lara LAS, Abdo CHN. Age at Time of Initial Sexual Intercourse and Health of Adolescent Girls. J Pediatr Adolesc Gynecol. 2016;29(5):417–23. 10.1016/j.jpag.2015.11.012 . 26655691

[5] Palmer RH, Young SE, Hopfer CJ, Corley RP, Stallings MC, Crowley TJ, et al Developmental epidemiology of drug use and abuse in adolescence and young adulthood: Evidence of generalized risk. Drug Alcohol Depend. 2009;102(1–3):78–87. 10.1016/j.drugalcdep.2009.01.012 19250776 PMC2746112

[6] Bauman A, Phongsavan P. Epidemiology of substance use in adolescence: prevalence, trends and policy implications. Drug Alcohol Depend. 1999;55(3):187–207. . 10428361 10.1016/s0376-8716(99)00016-2

[7] World Health Organisation. WHO report on the global tobacco epidemic, 2017: monitoring tobacco use and prevention policies. Geneva: World Health Organization; 2017.

[8] United Nations Office on Drugs and Crime. World Drug Report 2017. Vienna, Austria: United Nations publication; 2017.

[9] World Health Organisation. Global Status Report on Alcohol and Health. Geneva, Switzerland: World Health Organization, 2014.

[10] Word Health Organisation. Alcohol Use and Sexual Risk Behaviour: A Cross-Cultural Study in Eight Countries. Geneva, Switzerland: World Health Organisation, 2005.

[11] Francis JM, Weiss HA, Mshana G, Baisley K, Grosskurth H, Kapiga SH. The Epidemiology of Alcohol Use and Alcohol Use Disorders among Young People in Northern Tanzania. PloS one. 2015;10(10):e0140041 10.1371/journal.pone.0140041 26444441 PMC4596556

[12] Francis JM, Grosskurth H, Changalucha J, Kapiga SH, Weiss HA. Systematic review and meta-analysis: prevalence of alcohol use among young people in eastern Africa. Tropical medicine & international health: TM & IH. 2014;19(4):476–88. 10.1111/tmi.12267 24479379 PMC4065366

[13] Morojele N, Myers B., Townsend L., Lombard C., Plüddemann A., Carney T., et al Survey on Substance Use, Risk Behaviour and Mental Health among Grade 8–10 Learners in Western Cape Provincial Schools, 2011. Cape Town, SA: South African Medical Research Council, 2013 2013. Report No.

[14] Reddy SP, James S., Sewpaul R., Sifunda S., Ellahebokus A., Kambaran N.S., et al Umthente Uhlaba Usamila–The 3RD South African National Youth Risk Behaviour Survey 2011. Cape Town, South Africa: South African Medical Research Council, 2013.

[15] Patton GC, Sawyer SM, Santelli JS, Ross DA, Afifi R, Allen NB, et al Our future: a Lancet commission on adolescent health and wellbeing. Lancet. 2016;387(10036):2423–78. 10.1016/S0140-6736(16)00579-1 27174304 PMC5832967

[16] Degenhardt L, Stockings E, Patton G, Hall WD, Lynskey M. The increasing global health priority of substance use in young people. Lancet Psychiatry. 2016;3(3):251–64. 10.1016/S2215-0366(15)00508-8 . 26905480

[17] Peltzer K, Ramlagan S, Johnson BD, Phaswana-Mafuya N. Illicit drug use and treatment in South Africa: a review. Subst Use Misuse. 2010;45(13):2221–43. 10.3109/10826084.2010.481594 21039113 PMC3010753

[18] Ponte SR S.;Van Sittert L. Black Economic Empowerment’, Business and the State in South Africa. Development and Change. 2007;38(5):933–55.

[19] Christopher AJ. Urban Segregation in Post-apartheid South Africa. Urban Studies. 2016;38(3):449–66. 10.1080/00420980120080031

[20] Pinnock D. Gang Town. Cape Town: NB Publishers; 2016.

[21] Ward CM, T.; Bray R. Parenting, poverty and young people in South Africa: What are the connections? South African Child Gauge. 2015:70–4.

[22] Aziz G. Youth ministry as an agency of youth development for the vulnerable youth of the Cape Flats. Verbum et Ecclesia. 2017;38(1):a1745 10.4102/ve.v38i1.1745

[23] Masuku TM. Prophetic Mission of Faith Communities during Apartheid South Africa, 1948–1994: An agenda for Prophetic Mission Praxis in the Democratic SA. Missionalia. 2015;42(3):151–67. 10.7832/42-3-66

[24] Ganiel G. Pentecostal and charismatic Christianity in South Africa and Zimbabwe: A review. Religion Compass. 2010;4:130–43.

[25] AbdAleati NS, Mohd Zaharim N, Mydin YO. Religiousness and Mental Health: Systematic Review Study. Journal of religion and health. 2016;55(6):1929–37. 10.1007/s10943-014-9896-1 . 27654836

[26] Aukst-Margetic B, Margetic B. Religiosity and health outcomes: review of literature. Coll Antropol. 2005;29(1):365–71. . 16117349

[27] Cotton S, Berry D. Religiosity, spirituality, and adolescent sexuality. Adolescent medicine: state of the art reviews. 2007;18(3):471–83, vi. Epub 2008/05/06. . 18453228

[28] Gyimah SO, Kodzi I, Emina J, Cofie N, Ezeh A. Religion, religiosity and premarital sexual attitudes of young people in the informal settlements of Nairobi, Kenya. Journal of biosocial science. 2013;45(1):13–29. Epub 2012/06/22. 10.1017/S0021932012000168 . 22716919

[29] Kub J, Solari-Twadell PA. Religiosity/spirituality and substance use in adolescence as related to positive development: a literature review. J Addict Nurs. 2013;24(4):247–62. 10.1097/JAN.0000000000000006 . 24335772

[30] Moreau C, Trussell J, Bajos N. Religiosity, religious affiliation, and patterns of sexual activity and contraceptive use in France. The European journal of contraception & reproductive health care: the official journal of the European Society of Contraception. 2013;18(3):168–80. Epub 2013/04/04. 10.3109/13625187.2013.777829 23547890 PMC3656140

[31] Moreira-Almeida A, Neto FL, Koenig HG. Religiousness and mental health: a review. Rev Bras Psiquiatr. 2006;28(3):242–50. . 16924349 10.1590/s1516-44462006000300018

[32] Peltzer K, Pengpid S, Amuleru-Marshall O, Mufune P, Zeid AA. Religiosity and Health Risk Behaviour Among University Students in 26 Low, Middle and High Income Countries. Journal of religion and health. 2016;55(6):2131–40. Epub 2016/05/28. 10.1007/s10943-016-0260-5 . 27229939

[33] Rew L, Wong YJ. A systematic review of associations among religiosity/spirituality and adolescent health attitudes and behaviors. J Adolesc Health. 2006;38(4):433–42. 10.1016/j.jadohealth.2005.02.004 . 16549305

[34] Wong YJ, Rew L, Slaikeu KD. A systematic review of recent research on adolescent religiosity/spirituality and mental health. Issues Ment Health Nurs. 2006;27(2):161–83. 10.1080/01612840500436941 . 16418077

[35] VanderWeele TJ, Yu J, Cozier YC, Wise L, Argentieri MA, Rosenberg L, et al Attendance at Religious Services, Prayer, Religious Coping, and Religious/Spiritual Identity as Predictors of All-Cause Mortality in the Black Women's Health Study. Am J Epidemiol. 2017;185(7):515–22. 10.1093/aje/kww179 . 28338863 PMC6354668

[36] Yonker JE, Schnabelrauch CA, Dehaan LG. The relationship between spirituality and religiosity on psychological outcomes in adolescents and emerging adults: a meta-analytic review. J Adolesc. 2012;35(2):299–314. 10.1016/j.adolescence.2011.08.010 . 21920596

[37] Foster DW, Quist MC, Young CM, Bryan JL, Nguyen ML, Neighbors C. Benefit finding as a moderator of the relationship between spirituality/religiosity and drinking. Addict Behav. 2013;38(11):2647–52. 10.1016/j.addbeh.2013.06.019 23899427 PMC4214151

[38] Koenig HG. Religion, spirituality, and health: the research and clinical implications. ISRN Psychiatry. 2012;2012:278730 10.5402/2012/278730 23762764 PMC3671693

[39] Peres MFP, Kamei HH, Tobo PR, Lucchetti G. Mechanisms Behind Religiosity and Spirituality's Effect on Mental Health, Quality of Life and Well-Being. Journal of religion and health. 2018;57(5):1842–55. 10.1007/s10943-017-0400-6 . 28444608

[40] Greenfield EA, Marks NF. Religious Social Identity as an Explanatory Factor for Associations between More Frequent Formal Religious Participation and Psychological Well-Being. Int J Psychol Relig. 2007;17(3):245–59. 18698380 10.1080/10508610701402309 PMC2507864

[41] Rohrbaugh J, Jessor R. Religiosity in youth: a personal control against deviant behavior. J Pers. 1975;43(1):136–55. . 1142058 10.1111/j.1467-6494.1975.tb00577.x

[42] Storch EA, Strawser MS, Storch JB. Two-week test-retest reliability of the Duke Religion Index. Psychol Rep. 2004;94(3 Pt 1):993–4. 10.2466/pr0.94.3.993-994 . 15217062

[43] VanderWeele TJ. Causal effects of religious service attendance? Soc Psychiatry Psychiatr Epidemiol. 2017;52(11):1331–6. 10.1007/s00127-017-1434-5 . 28956085

[44] Muhwava LS, Morojele N, London L. Psychosocial factors associated with early initiation and frequency of antenatal care (ANC) visits in a rural and urban setting in South Africa: a cross-sectional survey. BMC Pregnancy Childbirth. 2016;16:18 10.1186/s12884-016-0807-1 26810320 PMC4727269

[45] Peltzer K, Pengpid S. Drinking and Driving among University Students in 22 Low, Middle Income and Emerging Economy Countries. Iran J Public Health. 2015;44(10):1330–8. 26576345 PMC4644577

[46] Heeren GA, Icard LD, O'Leary A, Jemmott JB, 3rd, Ngwane Z, Mtose X. Protective factors and HIV risk behavior among South African men. AIDS Behav. 2014;18(10):1991–7. 10.1007/s10461-014-0767-2 24722765 PMC4174730

[47] Viljoen D, Croxford J, Gossage JP, Kodituwakku PW, May PA. Characteristics of mothers of children with fetal alcohol syndrome in the Western Cape Province of South Africa: a case control study. J Stud Alcohol. 2002;63(1):6–17. . 11925060

[48] Parry CD, Morojele NK, Saban A, Flisher AJ. Brief report: Social and neighbourhood correlates of adolescent drunkenness: a pilot study in Cape Town, South Africa. J Adolesc. 2004;27(3):369–74. 10.1016/j.adolescence.2003.09.005 . 15159095

[49] Stats SA 2012 South African statistics 2012. Pretoria, South Africa: 2012.

[50] Stats SA 2017 Victims of Crime Survey 2015/16. Pretoria, SA: 2017.

[51] Peltzer K, Phaswana-Mafuya N. Drug use among youth and adults in a population-based survey in South Africa. S Afr J Psychiatr. 2018;24(0):1139 10.4102/sajpsychiatry.v24i0.1139 30263218 PMC6138152

[52] Shisana OR T.; Simbayi L.C.; Zuma K.; Jooste S.; Zungu N.;Labadarios D.; et al South African National HIV Prevalence, Incidence and Behaviour Survey, 2012. Cape Town: 2014. 10.2989/16085906.2016.1153491 27002359

[53] Pluddemann A, Flisher AJ, Mathews C, Carney T, Lombard C. Adolescent methamphetamine use and sexual risk behaviour in secondary school students in Cape Town, South Africa. Drug Alcohol Rev. 2008;27(6):687–92. 10.1080/09595230802245253 . 18825548

[54] Pluddemann A, Flisher AJ, McKetin R, Parry C, Lombard C. Methamphetamine use, aggressive behavior and other mental health issues among high-school students in Cape Town, South Africa. Drug Alcohol Depend. 2010;109(1–3):14–9. 10.1016/j.drugalcdep.2009.11.021 20064699 PMC3784347

[55] Sommer J, Hinsberger M, Elbert T, Holtzhausen L, Kaminer D, Seedat S, et al The interplay between trauma, substance abuse and appetitive aggression and its relation to criminal activity among high-risk males in South Africa. Addict Behav. 2017;64:29–34. 10.1016/j.addbeh.2016.08.008 27540760 PMC5102240

[56] Carney T, Myers BJ, Louw J, Lombard C, Flisher AJ. The relationship between substance use and delinquency among high-school students in Cape Town, South Africa. J Adolesc. 2013;36(3):447–55. 10.1016/j.adolescence.2013.01.004 . 23453849

[57] Saban A, Flisher AJ. The association between psychopathology and substance use in young people: a review of the literature. J Psychoactive Drugs. 2010;42(1):37–47. 10.1080/02791072.2010.10399784 . 20464805

[58] Nicholas LJ. The association between religiosity, sexual fantasy, participation in sexual acts, sexual enjoyment, exposure, and reaction to sexual materials among black South Africans. J Sex Marital Ther. 2004;30(1):37–42. 10.1080/00926230490247264 . 14660292

[59] Jabbour S, Fouad FM. Religion-based tobacco control interventions: how should WHO proceed? Bull World Health Organ. 2004;82(12):923–7. doi: /S0042-96862004001200008 15654406 PMC2623096

[60] Griffith DM, Campbell B, Allen JO, Robinson KJ, Stewart SK. YOUR Blessed Health: an HIV-prevention program bridging faith and public health communities. Public Health Rep. 2010;125 Suppl 1:4–11. 10.1177/00333549101250S102 20408382 PMC2788403

[61] Kuntsche S, Kuntsche E. Parent-based interventions for preventing or reducing adolescent substance use—A systematic literature review. Clin Psychol Rev. 2016;45:89–101. 10.1016/j.cpr.2016.02.004 . 27111301

